# Calcilytic NPSP795 Increases Plasma Calcium and PTH in an Autosomal Dominant Hypocalcemia Type 1 Mouse Model

**DOI:** 10.1002/jbm4.10402

**Published:** 2020-09-07

**Authors:** Fadil M Hannan, Caroline M Gorvin, Valerie N Babinsky, Mie K Olesen, Michelle Stewart, Sara Wells, Roger D Cox, Edward F Nemeth, Rajesh V Thakker

**Affiliations:** ^1^ Academic Endocrine Unit, Radcliffe Department of Medicine Oxford Centre for Diabetes, Endocrinology and Metabolism (OCDEM), University of Oxford Oxford UK; ^2^ MRC Mammalian Genetics Unit and Mary Lyon Centre MRC Harwell Institute, Harwell Science and Innovation Campus Oxford UK; ^3^ MetisMedica Toronto Canada

**Keywords:** ENDOCRINE PATHWAYS, GENETIC ANIMAL MODELS, HORMONE REPLACEMENT/RECEPTOR MODULATORS, PARATHYROID‐RELATED DISORDERS

## Abstract

Calcilytics are calcium‐sensing receptor (CaSR) antagonists that reduce the sensitivity of the CaSR to extracellular calcium. Calcilytics have the potential to treat autosomal dominant hypocalcemia type 1 (ADH1), which is caused by germline gain‐of‐function CaSR mutations and leads to symptomatic hypocalcemia, inappropriately low PTH concentrations, and hypercalciuria. To date, only one calcilytic compound, NPSP795, has been evaluated in patients with ADH1: Doses of up to 30 mg per patient have been shown to increase PTH concentrations, but did not significantly alter ionized blood calcium concentrations. The aim of this study was to further investigate NPSP795 for the treatment of ADH1 by undertaking in vitro and in vivo studies involving *Nuf* mice, which have hypocalcemia in association with a gain‐of‐function CaSR mutation, Leu723Gln. Treatment of HEK293 cells stably expressing the mutant *Nuf* (Gln723) CaSR with 20nM NPSP795 decreased extracellular Ca^2+^‐mediated intracellular calcium and phosphorylated ERK responses. An in vivo dose‐ranging study was undertaken by administering a s.c. bolus of NPSP795 at doses ranging from 0 to 30 mg/kg to heterozygous *(Casr* ^*+/Nuf*^ *)* and to homozygous *(Casr* ^*Nuf/Nuf*^ *)* mice, and measuring plasma PTH responses at 30 min postdose. NPSP795 significantly increased plasma PTH concentrations in a dose‐dependent manner with the 30 mg/kg dose causing a maximal (≥10‐fold) rise in PTH. To determine whether NPSP795 can rectify the hypocalcemia of *Casr* ^*+/Nuf*^ and *Casr* ^*Nuf/Nuf*^ mice, a submaximal dose (25 mg/kg) was administered, and plasma adjusted‐calcium concentrations measured over a 6‐hour period. NPSP795 significantly increased plasma adjusted‐calcium in *Casr* ^*+/Nuf*^ mice from 1.87 ± 0.03 mmol/L to 2.16 ± 0.06 mmol/L, and in *Casr* ^*Nuf/Nuf*^ mice from 1.70 ± 0.03 mmol/L to 1.89 ± 0.05 mmol/L. Our findings show that NPSP795 elicits dose‐dependent increases in PTH and ameliorates the hypocalcemia in an ADH1 mouse model. Thus, calcilytics such as NPSP795 represent a potential targeted therapy for ADH1. © 2020 The Authors. *JBMR Plus* published by Wiley Periodicals LLC on behalf of American Society for Bone and Mineral Research.

## Introduction

Autosomal dominant hypocalcemia (ADH) is a genetically heterogeneous disorder of extracellular calcium (Ca^2+^_e_) homeostasis consisting of two reported variants: ADH type 1 (ADH1; OMIM #601198) is caused by germline gain‐of‐function mutations of the G‐protein‐coupled calcium‐sensing receptor (CaSR),^(^ ^1^, ^2^ ^)^ and ADH type 2 (ADH2; OMIM #615361) is caused by germline gain‐of‐function mutations of G‐protein subunit α‐11 (Gα_11_).^(^ ^3^, ^4^, ^5^, ^6^ ^)^ The CaSR and Gα_11_ proteins signal via multiple pathways, including intracellular calcium (Ca^2+^_i_) mobilization and the ERK arm of the MAPK cascade to regulate PTH secretion and urinary calcium excretion.^(^ ^7^, ^8^ ^)^ ADH1 is the most common disease variant, with an estimated prevalence of 3.9 cases per 100,000,^(^ ^9^ ^)^ and is characterized by hypocalcemia, increased circulating phosphate concentrations, inappropriately low or normal PTH concentrations, and a relative hypercalciuria with urinary calcium‐to‐creatinine ratios that are within or above the reference range.^(^ ^1^, ^3^, ^10^, ^11^ ^)^ ADH1 has a substantial burden of illness and causes hypocalcemic symptoms such as paraesthesia, muscle spasms, and seizures in around 50% of patients.^(^ ^10^ ^)^ Ectopic calcifications are also common in ADH1 and >35% of patients develop basal ganglia calcifications, whereas >10% of patients have nephrocalcinosis.^(^ ^10^ ^)^ Furthermore, patients with severe forms of ADH1 can develop a Bartter‐like syndrome, characterized by hypokalemic alkalosis, renal salt wasting, and hyperreninemic hyperaldosteronism.^(^ ^12^, ^13^ ^)^

ADH1 has a high unmet clinical need as conventional therapies such as vitamin D analogs (eg, alfacalcidol and calcitriol) and calcium supplements predispose patients with ADH1 to the development of marked hypercalciuria, nephrocalcinosis, nephrolithiasis, and renal failure.^(^ ^1^, ^10^ ^)^ Recombinant PTH injections have occasionally been used to treat symptomatic forms of ADH1.^(^ ^14^ ^)^ However, use of this treatment is expensive and limited, as it is administered by s.c. bolus injections or continuous pump infusion, and may not prevent patients with ADH1 from developing hypercalciuric renal complications.^(^ ^14^ ^)^ Thus, better treatments are required, and antagonists of the CaSR, which are referred to as calcilytics,^(^ ^15^, ^16^ ^)^ have the potential to act as a targeted therapy for ADH1. To date, all calcilytics are negative allosteric modulators (NAMs) of the CaSR and comprise two main classes of orally active compounds: the amino‐alcohols and the quinazolinones. Calcilytics were originally investigated as therapies for osteoporosis, as these compounds transiently stimulated PTH secretion, which had the potential to induce bone anabolic effects.^(^ ^17^ ^)^ However, clinical trials have shown that calcilytics have a lack of efficacy for postmenopausal osteoporosis,^(^ ^18^, ^19^ ^)^ but can lead to sustained elevations in serum calcium concentrations in healthy subjects,^(^ ^20^, ^21^ ^)^ thereby highlighting the potential of CaSR NAMs to treat hypocalcemic disorders such as ADH. In support of this, calcilytics such as NPS 2143, NPSP795, JTT‐305/MK‐5442, and AXT914 have been shown to normalize the increased signaling responses associated with ADH1‐causing mutant CaSRs in vitro,^(^ ^22^, ^23^, ^24^, ^25^ ^)^ and the calcilytics NPS 2143 and JTT‐305/MK‐5442 have also been shown to increase plasma calcium and PTH concentrations in ADH1 mouse models in vivo.^(^ ^24^, ^26^ ^)^ However, the effectiveness of calcilytics as treatments for patients with ADH1 remains unclear. For example, a phase IIb study involving 5 patients with ADH1 showed that i.v. administration of NPSP795 (an amino‐alcohol calcilytic compound) at doses ranging from 5 to 30 mg, increased PTH, but did not significantly alter ionized blood calcium concentrations.^(^ ^23^ ^)^ We have further evaluated the efficacy of NPSP795 treatment for ADH1 by undertaking in vitro and in vivo studies involving *Nuf* mice, which have hypocalcemia (Table 1) in association with a germline gain‐of‐function CaSR mutation, Leu723Gln. Our findings demonstrate that NPSP795 increases PTH in a dose‐dependent manner, and that a higher dose (25 mg/kg) than that used in the reported phase IIb study,^(^ ^23^ ^)^ significantly increases plasma calcium concentrations.

**Table 1:** Age and Plasma Biochemistry of WT, *Casr* ^*+/Nuf*^, and *Casr* ^*Nuf/Nuf*^ Mice^a^

	Male			Female		
	*+/+*	*+/Nuf*	*Nuf/Nuf*	*+/+*	*+/Nuf*	*Nuf/Nuf*
	*n* = 6	*n* = 5	*n* = 6	*n* = 5	*n* = 6	*n* = 5
Age (weeks)	30.1 ± 0.9	29.4 ± 0.7	28.3 ± 1.4	28.7 ± 1.1	29.5 ± 0.5	31.0 ± 1.6
Tot‐Ca (mmol/L)	2.40 ± 0.05	1.82 ± 0.03**	1.71 ± 0.03**	2.53 ± 0.02	1.89 ± 0.05**	1.73 ± 0.05**
Albumin (g/L)	28.4 ± 0.7	29.1 ± 0.5	28.5 ± 0.2	30.6 ± 1.2	29.8 ± 1.3	29.9 ± 0.7
Adj‐Ca (mmol/L)	2.43 ± 0.04	1.83 ± 0.03**	1.74 ± 0.03**	2.52 ± 0.03	1.89 ± 0.03**	1.73 ± 0.05**
Phosphate (mmol/L)	1.39 ± 0.09	2.09 ± 0.21	2.26 ± 0.3*	1.10 ± 0.15	1.79 ± 0.2	2.19 ± 0.19*
ALP (U/L)	62.7 ± 10.8	76.6 ± 4.5	60.3 ± 9.1	126 ± 13	77.8 ± 7.4*	99.5 ± 11
PTH (ng/L)	89.7 ± 16	12.9 ± 3.0**	3.0 ± 1.5**	51.7 ± 16	16.8 ± 4.5*	7.6 ± 3.0*
Urea (mmol/L)	9.6 ± 0.8	9.6 ± 0.7	10.8 ± 0.7	9.0 ± 0.7	9.8 ± 0.9	9.9 ± 1.3
Creatinine (μmol/L)	11.1 ± 0.9	12.0 ± 0.8	11.6 ± 0.6	15.9 ± 1.4	16.2 ± 1.2	15.7 ± 1.3

Adj‐Ca = Albumin‐adjusted calcium; ALP, alkaline phosphatase activity; Tot‐Ca = total calcium.

^a^ Age and biochemical parameters of untreated male and female WT (*+/+*), *Casr* ^*+/Nuf*^ *(+/Nuf)*, and *Casr* ^*Nuf/Nuf*^ *(Nuf/Nuf)* mice are given. All data are shown as mean ± SEM.

* *p* < 0.05 compared with WT (*+/+*) mice.

** *p* < 0.001 compared with WT (*+/+*) mice.

## Materials and Methods

### Compounds

NPSP795, which is also known as SHP635, was provided by NPS/Shire Pharmaceuticals (Lexington, MA, USA) and dissolved in a 20% aqueous solution of 2‐hydroxypropyl‐β‐cyclodextrin (Sigma‐Aldrich, St. Louis, MO, USA) prior to use in in vitro and in vivo studies.

### Animals

All study mice were littermates aged between 22 to 31 weeks. Mice were kept in accordance with welfare guidance from the UK Government Home Office Department (London, UK) in an environment controlled for light (12‐hour light/dark cycle), temperature (21 ± 2°C), and humidity (55% ± 10%) at the Medical Research Council (MRC) Harwell Centre (Oxfordshire, UK).^(^ ^27^ ^)^ Mice had free access to water (25 ppm chlorine) and were fed *ad libitum* a commercial diet (RM3; Special Diet Services, Witham, UK) that contained 1.24% calcium, 0.83% phosphorus, and 2948 IU/kg of vitamin D. *Nuf* mice (MGI ID: MGI:3054788) were maintained on the inbred 102/H strain background (strain ID: C3;102‐CasrNuf/H; MGI:5291924), which is a substrain bred at the Mary Lyon Centre (Harwell, UK).^(^ ^26^, ^28^ ^)^ Animal studies were approved by the MRC Harwell Institute Ethical Review Committee, and were licensed under the Animal (Scientific Procedures) Act 1986, issued by the UK Government Home Office Department (PPL30/2752).

### Generation of cells stably expressing the CaSR

HEK293 cells stably expressing the WT and mutant CaSR (TRex‐CaSR‐WT and TRex‐CaSR‐Gln723, respectively) were generated using the TRex‐HEK293 Flp‐In cell‐line system (Invitrogen).^(^ ^29^, ^30^ ^)^ WT CaSR was subcloned in‐frame from a pcDNA3.1+ construct^(^ ^1^ ^)^ into a pcDNA5/FRT/TO construct (Invitrogen, Carlsbad, CA, USA) using the *HindIII* and *ApaI* restriction sites. The mutant CaSR construct was generated by site‐directed mutagenesis using the Quikchange Lightning kit (Agilent Technologies, Santa Clara, CA, USA).^(^ ^3^ ^)^ Mutagenesis and cloning were confirmed using gene‐specific primers (Sigma‐Aldrich) and DNA sequence analysis, as reported.^(^ ^3^ ^)^ TRex‐HEK293 cells were maintained in DMEM Glutamax medium (Gibco, Grand Island, NY, USA) supplemented with 10% FBS (Gibco), 2mM glutamine (Gibco), 15 μg/mL blasticidin (Invitrogen), and 100 μg/mL zeocin (Invitrogen). Cells were transfected with 1 μg of either WT or mutant CaSR alongside the pOG44 Flp‐recombinase expression vector (Invitrogen) using lipofectamine 2000 (Invitrogen).^(^ ^29^ ^)^ Cells expressing the constructs were selected using 100 μg/mL hygromycin B (Invitrogen). Colonies of hygromycin‐resistant cells were picked, expanded, and expression of CaSR protein was tested in each clonal cell population by Western blot analysis using an anti‐CaSR antibody (Abcam, Cambridge, UK), as described.^(^ ^3^ ^)^ Calnexin expression was used as a loading control and detected using an anticalnexin antibody (Millipore, Watford, UK). Blots were visualized using an Immuno‐Star WesternC kit (Bio‐Rad Laboratories, Hercules, CA, USA) on a BioRad Chemidoc XRS+ system.^(^ ^6^ ^)^ Twenty‐four hours prior to performance of in vitro assays, expression of the CaSR protein was induced using 1 μg/mL tetracycline (Invitrogen).

### Intracellular calcium measurements

The Ca^2+^_i_ responses of TRex‐CaSR‐WT and mutant TRex‐CaSR‐Gln723 cells were assessed by a flow cytometry‐based assay, as reported.^(^ ^2^, ^3^ ^)^ Briefly, 48 hours posttransfection, the cells were harvested, washed in calcium‐ and magnesium‐free Hank's balanced salt solution (HBSS; Invitrogen), and loaded with 1 μg/mL indo‐1‐acetoxymethylester (Indo‐1 AM) (Molecular Probes, Eugene, OR, USA) for 1 hour at 37°C.^(^ ^2^, ^3^ ^)^ After the removal of free dye, the cells were resuspended in calcium‐ and magnesium‐free HBSS and maintained at 37°C. TRex‐CaSR‐WT and mutant TRex‐CaSR‐Gln723 cells were incubated with either a 20% aqueous solution of 2‐hydoxypropyl‐β‐cyclodextrin (vehicle) or NPSP795 at concentrations of 20 and 40nM for 1 hour, as described.^(^ ^31^ ^)^ Cells in suspension were stimulated by sequentially adding calcium to increase the Ca^2+^_e_ concentration in a stepwise manner from 0 to 15mM, and then analyzed on a MoFlo modular flow cytometer (Beckman Coulter, Brea, CA, USA) by measurement of Ca^2+^_i_‐bound Indo‐1 am (at 410 nm), and free Indo‐1 am (at 485 nm), using a JDSU Xcyte UV laser (Coherent, Inc., Santa Clara, CA, USA), on each cell at each Ca^2+^_e_ concentration, as described.^(^ ^2^, ^3^ ^)^ Cytomation Summit software (Beckman Coulter) was used to determine the peak mean fluorescence ratio of the transient response after each individual stimulus and expressed as a normalized response.^(^ ^2^, ^3^ ^)^ Concentration‐response curves were generated using a four‐parameter nonlinear regression curve‐fit model (GraphPad Prism; GraphPad Software, Inc., La Jolla, CA, USA) to calculate the half‐maximal (EC_50_) values.^(^ ^2^, ^3^ ^)^

### Phosphorylated and total ERK measurements

TRex‐CaSR‐WT and mutant TRex‐CaSR‐Gln723 cells were seeded in poly‐L‐lysine treated 48‐well plates and incubated for 24 hours. The following day, the medium was changed to serum‐free tetracycline selection medium and incubated for a further 12 hours prior to treatment of cells with 0 to 10mM CaCl_2_. Cells were lysed in Surefire lysis buffer, and AlphaScreen Surefire ERK assays (PerkinElmer, Waltham, MA, USA) measuring phosphorylated and total proteins performed, as described.^(^ ^6^, ^31^ ^)^ For studies with NPSP795, cells were incubated with either a 20% aqueous solution of 2‐hydoxypropyl‐β‐cyclodextrin (vehicle) or NPSP795 for 4 hours prior to calcium treatment. The fluorescence signal in both assays was measured using the PheraStar FS microplate reader (BMG Labtech, Ortenberg, Germany).^(^ ^6^, ^31^ ^)^ Fold‐change phosphorylated ERK (pERK) responses were expressed as a ratio of pERK to total ERK responses.

### In vivo administration of NPSP795 to *Nuf* mice

Mice were randomly allocated to receive NPSP795 or vehicle as a single bolus by s.c. injection. None of the mice had undergone any experimental procedures prior to dosing. Study investigators were blinded during animal handling, and also when undertaking endpoint measurements. The primary experimental outcome was a change in plasma calcium at 1‐hour postdose in heterozygous *(Casr* ^*+/Nuf*^ *)* mice. Blood samples were collected from the lateral tail vein following application of topical local anesthesia for measurement of plasma PTH, or collected from the retro‐orbital vein under isoflurane terminal anesthesia for measurement of other plasma biochemical parameters.^(^ ^26^, ^27^ ^)^

### Plasma biochemical analyses

Plasma was separated by centrifugation at 5000*g* for 10 min at 8°C, and analyzed for calcium, albumin, phosphate, urea, and creatinine on a Beckman Coulter AU680 analyzer, as described.^(^ ^26^ ^)^ Plasma calcium was adjusted for variations in albumin concentrations using the formula: (plasma calcium (mmol/L) – ([plasma albumin [g/L] – 30] × 0.02], as reported.^(^ ^27^ ^)^ Plasma PTH concentrations were determined using an ELISA for mouse intact PTH (Immutopics, Inc., San Clemente, CA, USA).^(^ ^26^ ^)^ Fold‐change PTH responses are expressed as a ratio of plasma PTH concentrations of NPSP795‐treated mice to the mean plasma PTH concentrations of respective vehicle‐treated mice.

### Statistical analyses

All in vitro studies were performed in four biological replicates. For the in vitro measurement of Ca^2+^_i_ responses, statistical comparisons were undertaken using the *F* test.^(^ ^2^, ^3^ ^)^ Fold‐change pERK responses were analyzed by two‐way ANOVA with Tukey's multiple‐comparisons test. Mouse sample size calculations were undertaken using G*Power statistical software. The unit of analysis was a single mouse. A sample size of *n* = 5 mice allocated to the treatment and control groups provided >80% power to detect a >15% increase in plasma calcium concentrations. Biochemical parameters were analyzed by one‐way ANOVA with Sidak's multiple‐comparisons test. All analyses were undertaken using GraphPad Prism (GraphPad), and a value of *p* < 0.05 was considered significant. All data are shown as mean ± SEM.

## Results

### Effect of NPSP795 on the signaling responses of cells expressing the gain‐of‐function mutant Gln723 CaSR

**Fig 1:**
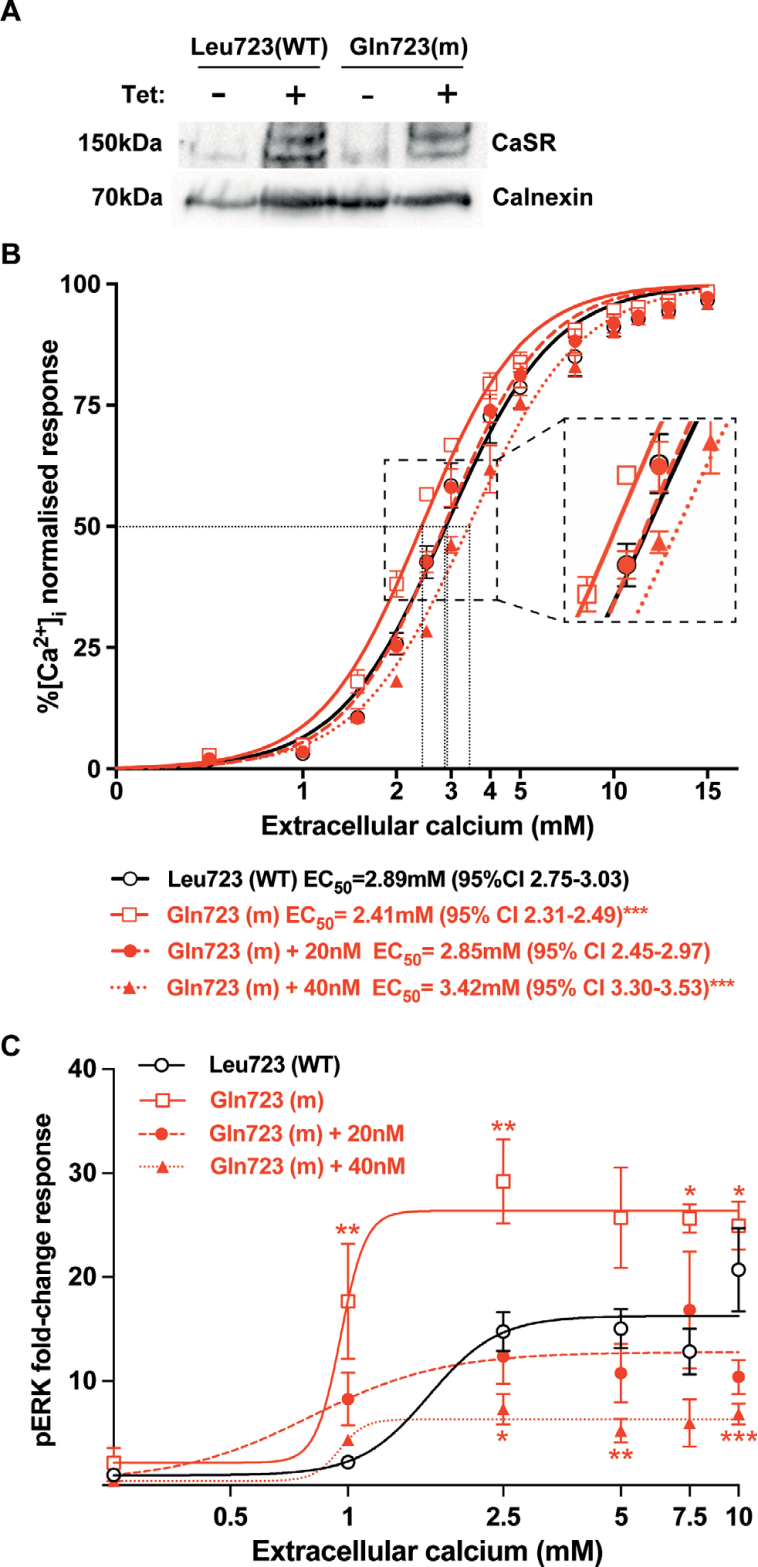
Effect of NPSP795 on the signaling responses of cells stably expressing the WT or mutant Gln723 CaSR. (*A*) Western blot analysis of lysates from TRex‐CaSR‐WT and mutant TRex‐CaSR‐Gln723 cells demonstrating that addition of tetracycline (Tet) induces increased CaSR expression. Calnexin was used as a housekeeping protein. (*B*) Ca^2+^_i_ response to changes in Ca^2+^_e_ concentrations of TRex‐CaSR‐WT and mutant TRex‐CaSR‐Gln723 cells. The Ca^2+^_i_ responses to changes in Ca^2+^_e_ concentrations are expressed as a percentage of the maximum normalized responses. The Gln723 CaSR mutant led to a leftward shift in the concentration‐response curve (solid‐red line). The addition of 20nM NPSP795 rectified the leftward shift of the Gln723 CaSR mutant (dashed‐red line), whereas 40nM NPSP795 caused a rightward shift of the Gln723 CaSR mutant (dotted‐red line) compared with WT (solid‐black line). Dotted black lines indicate the EC_50_ values for respective WT and mutant cells. A close‐up view of the concentration‐response curves at the half‐maximal response is also shown. (*C*) pERK fold‐change response to changes in Ca^2+^_e_ concentrations of TRex‐CaSR‐WT and mutant TRex‐CaSR‐Gln723 cells. The fold‐change responses of pERK are expressed as a ratio of the total ERK response. The Gln723 mutant (solid‐red line) led to significantly increased pERK fold‐change responses compared with WT (solid‐black line). The addition of 20nM NPSP795 normalized the pERK fold‐change responses of the Gln723 mutant (dashed‐red line), whereas 40nM NPSP795 significantly reduced pERK responses (dotted‐red line) compared with WT. *p < 0.05, **p < 0.01, ***p < 0.001 compared with WT.

To investigate the effect of NPSP795 on CaSR signal transduction, we established cells stably expressing WT CaSR or the *Nuf* mutant Gln723 CaSR, using the TRex Flp‐in system.^(^ ^29^, ^30^ ^)^ Following clonal cell selection, tetracycline addition to the culture media caused a robust overexpression of the CaSR protein in both WT and mutant Gln723 cells (Fig. 1*A*). We assessed whether NPSP795 could rectify alterations in Ca^2+^_e_‐mediated Ca^2+^_i_ responses in the mutant TRex‐CaSR‐Gln723 cells. As reported previously,^(^ ^26^, ^28^ ^)^ cells expressing the mutant Gln723 CaSR showed a leftward shift in the Ca^2+^_e_‐mediated Ca^2+^_i_ concentration‐response curve and a significantly reduced EC_50_ value (2.41 ± 0.05mM) when compared with the WT (Leu723) CaSR (EC_50_ = 2.89 ± 0.07mM; *p* < 0.001; Fig. 1*B*), consistent with a CaSR gain‐of‐function. A dose titration of NPSP795 in mutant Gln723 CaSR‐expressing cells showed that 20nM of NPSP795 normalized Ca^2+^_e_‐mediated Ca^2+^_i_ responses (EC_50_ = 2.85 ± 0.13mM), whereas 40nM of NPSP795 led to a rightward shift of the mutant receptor concentration‐response curve and significantly increased the EC_50_ value (3.42 ± 0.06mM; *p* < 0.001), compared with WT cells (Fig. 1*B*).

We also investigated the effect of NPSP795 on pERK responses in mutant Gln723 CaSR‐expressing cells following exposure to increasing Ca^2+^_e_ concentrations. The maximal fold‐changes in pERK responses of the untreated Gln723 CaSR mutant were shown to be significantly increased compared with WT cells (Gln723 = 24.9 ± 2.3 versus 20.7 ± 4.0 for WT; *p* < 0.05; Fig. 1*C*). Treatment of mutant Gln723 CaSR‐expressing cells with 20nM NPSP795 decreased the maximal pERK fold‐change response to 10.4 ± 1.7, which was not significantly different from WT (Fig. 1*C*). Thus, a 20nM dose of NPSP795 normalized the gain‐of‐function associated with the Leu723Gln CaSR mutation. In contrast, the addition of 40nM NPSP795 significantly reduced the mutant Gln723 CaSR pERK fold‐change response to 6.9 ± 1.0 (*p* < 0.001) compared with WT (Fig. 1*C*).

### Dose‐dependent effects of NPSP795 on plasma PTH in *Nuf* mice

**Fig 2:**
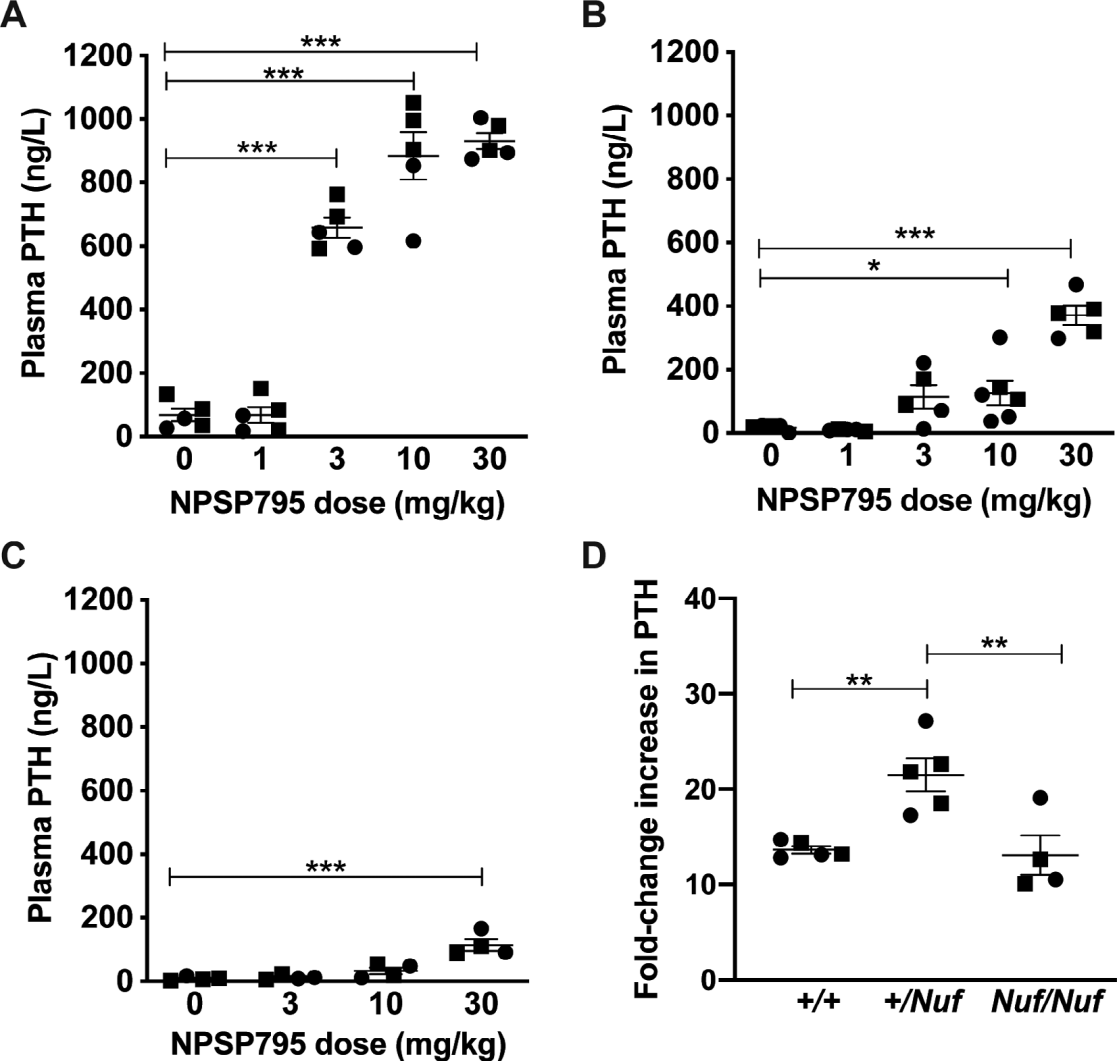
Dose‐dependent effects of NPSP795 on the plasma PTH responses of WT, *Casr* ^*+/Nuf*^, and *Casr* ^*Nuf/Nuf*^ mice. (*A–C*) Plasma PTH concentrations were measured in (*A*) WT, (*B*) *Casr* ^*+/Nuf*^, and (*C*) *Casr* ^*Nuf/Nuf*^ mice following administration of NPSP795 at doses ranging from 0 to 30 mg/kg. (*D*) Fold‐change plasma PTH responses following administration of 30 mg/kg NPSP795 to WT (*+/+*), *Casr* ^*+/Nuf*^ (*+/Nuf*), and *Casr* ^*Nuf/Nuf*^ (*Nuf/Nuf*) mice. Fold‐changes in plasma PTH are expressed as a ratio of the PTH values of NPSP795‐treated mice to the mean plasma PTH values of respective vehicle‐treated mice. Male and female mice are represented by squares and circles, respectively. Mean ± SEM values for the respective groups are indicated by solid bars. *p < 0.05, **p < 0.01, ***p < 0.001.

As NPSP795 rectified the altered signaling responses associated with the *Nuf* mouse CaSR mutation (Leu723Gln) in vitro, we pursued studies to determine the effects of this calcilytic in *Nuf* mice, which have hypocalcemia and reduced plasma PTH concentrations (Table 1). A dose‐ranging study was undertaken with NPSP795 to establish the doses required to maximally increase PTH concentrations. NPSP795 was administered at 0, 1, 3, 10, and 30 mg/kg doses by s.c. bolus injection to WT and *Casr* ^*+/Nuf*^ mice, and at 0, 3, 10, and 30 mg/kg doses to homozygous *(Casr* ^*Nuf/Nuf*^ *)* mice. A plasma sample was obtained at 30 min for PTH measurement. This time‐point was selected as plasma PTH concentrations have been reported to be maximally increased at 15 to 30 min following calcilytic administration in rats.^(^ ^32^ ^)^ NPSP795 administration to WT mice led to dose‐dependent increases in PTH concentrations, with 10 and 30 mg/kg doses causing maximal elevations of PTH (Fig. 2*A*). NPSP795 administration also caused dose‐dependent PTH elevations in *Casr* ^*+/Nuf*^ and *Casr* ^*Nuf/Nuf*^ mice, although higher calcilytic doses were required to increase PTH in mutant mice (Fig. 2*B*,*C*). Thus, *Casr* ^*+/Nuf*^ and *Casr* ^*Nuf/Nuf*^ mice required a minimum of 10 and 30 mg/kg of NPSP795, respectively, to significantly increase plasma PTH, compared with a minimum of 3 mg/kg for WT mice (Fig. 2*A*–*C*). *Casr* ^*+/Nuf*^ and *Casr* ^*Nuf/Nuf*^ mice treated with the highest (30 mg/kg) NPSP795 dose showed significantly reduced plasma PTH concentrations of 371 ± 30 ng/L and 114 ± 18 ng/L, respectively (*p* < 0.001), compared with a PTH concentration of 931 ± 26 ng/L for WT mice treated with the same dose. However, an analysis of fold‐change PTH responses at the 30 mg/kg dose showed that mutant mice have similar or increased PTH responses compared with WT mice (Fig. 2*D*). Thus, WT and *Casr* ^*Nuf/Nuf*^ mice all showed ≥10‐fold increases in plasma PTH compared with respective vehicle‐treated mice, whereas *Casr* ^*+/Nuf*^ mice showed significantly higher (>15‐fold) PTH responses (Fig. 2*D*).

### Time‐dependent effects of NPSP795 on plasma PTH, calcium, phosphate, urea, and creatinine in *Nuf* mice

**Fig 3:**
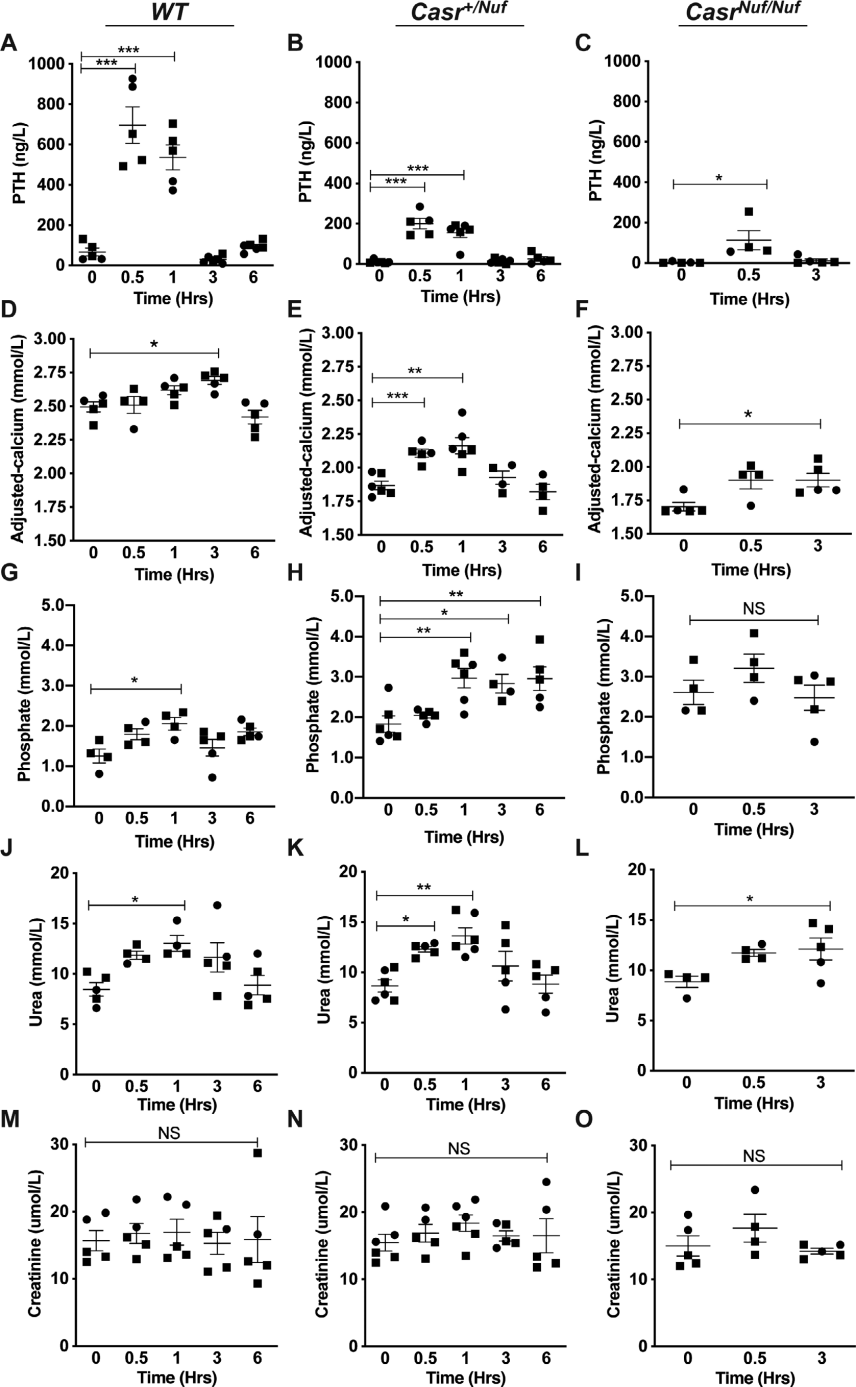
Time‐dependent effects of NPSP795 on plasma PTH, calcium, phosphate, urea, and creatinine concentrations in WT, *Casr* ^*+/Nuf*^, and *Casr* ^*Nuf/Nuf*^ mice. Plasma concentrations of (*A–C*) PTH, (*D–F*) adjusted calcium, (*G–I*) phosphate, (*J–L*) urea, and (*M–O*) creatinine are shown in WT and *Casr* ^*+/Nuf*^ mice at 0, 0.5, 1, 3, and 6 hours, and at 0, 0.5, and 3 hours in *Casr* ^*Nuf/Nuf*^ mice. Male and female mice are represented by squares and circles, respectively. Mean ± SEM values for the respective groups are indicated by solid bars. *p < 0.05, **p < 0.01, ***p < 0.001, NS, not significant.

To determine whether NPSP795 can rectify the hypocalcemia of *Nuf* mice (Table 1), a submaximal dose (25 mg/kg) was administered by s.c. bolus injection, and plasma concentrations of adjusted‐calcium, phosphate, PTH, urea, and creatinine measured at 0, 0.5, 1, 3, and 6 hours postdose in WT and *Casr* ^*+/Nuf*^ mice, and at 0, 0.5, and 3 hours in *Casr* ^*Nuf/Nuf*^ mice (Fig. 3). Administration of 25 mg/kg NPSP795 led to a maximal rise in plasma PTH concentrations at 30 min postdose, which returned to baseline values by 3 hours postdose in WT and *Nuf* mice (Fig. 3*A*–*C*). The rise in PTH was associated with significant elevations of plasma calcium at between 1 to 3 hours postdose in WT, *Casr* ^*+/Nuf*^, and *Casr* ^*Nuf/Nuf*^ mice when compared with respective untreated mice (Fig. 3*D*–*F*). Thus, NPSP795 significantly increased plasma calcium in *Casr* ^*+/Nuf*^ mice from 1.87 ± 0.03 to 2.16 ± 0.06 mmol/L, and in *Casr* ^*Nuf/Nuf*^ mice from 1.70 ± 0.03 to 1.89 ± 0.05 mmol/L (Fig. 3*E*,*F*). These increases in plasma calcium of between 0.20 and 0.30 mmol/L postdose were similar to that observed for WT mice treated with NPSP795 (Fig. 3*D*). Administration of this calcilytic also led to significant increases in plasma phosphate in WT and *Casr* ^*+/Nuf*^ mice (Fig. 3*G*–*I*). Single‐dose administration of NPSP795 was well‐tolerated by the study mice. However, an increase in plasma urea concentrations was observed (Fig. 3*J*–*L*), which was associated with normal plasma creatinine concentrations in WT, *Casr* ^*+/Nuf*^, and *Casr* ^*Nuf/Nuf*^ mice treated with NPSP795 (Fig. 3*M*–*O*). The sources of increased plasma urea with normal plasma creatinine include dehydration, heart failure, gastrointestinal bleeding, a high‐protein diet, and catabolic states caused by trauma, starvation, and the use of glucocorticoid drugs.^(^ ^33^ ^)^ Among these, the most likely cause was dehydration. Moreover, the rise in plasma urea was transient and had normalized at 6 hours postdose (Fig. 3*J*–*K*).

## Discussion

These findings demonstrate that the amino‐alcohol calcilytic, NPSP795, rectifies the gain‐of‐function associated with the *Nuf* mouse germline CaSR mutation, Leu723Gln,^(^ ^28^ ^)^ and increases plasma PTH and calcium concentrations in this ADH1 mouse model. We selected 20nM and 40nM NPSP795 concentrations for the cellular signaling studies as NPSP795 has a similar potency (IC_50_ = 73nM) to that of the NPS 2143 calcilytic compound (IC_50_ = 43nM),^(^ ^16^ ^)^ and our previous studies involving NPS 2143 have demonstrated these concentrations to significantly increase the Ca^2+^_i_ EC_50_ value of cells expressing the *Nuf* mutant CaSR.^(^ ^26^ ^)^ NPSP795 was shown to normalize increases in Ca^2+^_i_ and pERK‐signaling responses of cells stably expressing the *Nuf* mutant CaSR, which is in keeping with the reported effects of NPSP795 on ADH1‐causing germline gain‐of‐function CaSR mutations.^(^ ^23^ ^)^

Our in vivo studies showed that single‐bolus administration of NPSP795 significantly increases plasma PTH concentrations in a dose‐dependent manner in *Nuf* mice. However, substantially higher doses of NPSP795 were required to increase PTH secretion in *Casr* ^*+/Nuf*^ and *Casr* ^*Nuf/Nuf*^ mice compared with that required for WT mice (Fig. 2*A–C*). This was particularly evident for *Casr* ^*Nuf/Nuf*^ mice, which required 30 mg/kg NPSP795 to increase plasma PTH compared with 3 mg/kg of NPSP795 for WT mice. These findings suggest that parathyroid glands harboring the Gln723 mutant CaSR may have reduced sensitivity to NPSP795 compared with the parathyroid glands of WT mice. However, *Casr* ^*+/Nuf*^ and *Casr* ^*Nuf/Nuf*^ mice given the highest (30 mg/kg) NPSP795 dose had similar or increased fold‐change elevations in PTH responses compared with WT mice (Fig. 2*D*). These results are consistent with the biochemical features of ADH1 being rectifiable through normalization of the parathyroid set point for PTH release. Moreover, these findings differentiate ADH1 from hypoparathyroidism, which is generally associated with irreversible destruction of the parathyroid glands.^(^ ^34^ ^)^ Bolus dose administration of NPSP795 also significantly increased plasma calcium concentrations in *Casr* ^*+/Nuf*^ and *Casr* ^*Nuf/Nuf*^ mice, and the 0.2 to 0.3 mmol/L increase in plasma calcium was similar to that observed in WT mice treated with NPSP795 (Fig. 3). This finding contrasts with a reported clinical trial involving patients with ADH1, which observed no alterations in ionized blood calcium concentrations of patients with ADH1 given 5 to 30 mg of NPSP795 by i.v. administration.^(^ ^23^ ^)^ However, our study used a markedly higher (25 mg/kg) dose of this calcilytic, and the difference in dosing between the patient and mouse study likely explains the differences observed in circulating calcium responses. The plasma calcium concentrations of *Casr* ^*+/Nuf*^ mice treated with 25 mg/kg of NPSP795 remained significantly lower than that of untreated WT mice (2.16 ± 0.06 versus 2.50 ± 0.04 mmol/L; *p* < 0.01). However, we postulate that repetitive dosing with 25 mg/kg of NPSP795 will lead to normocalcemia in *Casr* ^*+/Nuf*^ mice, as this dose led to substantial (>15‐fold) elevations of plasma PTH (Fig. 3*B*). Consistent with this, a study reporting treatment of ADH1 mice with the JTT‐305/MK‐5442 calcilytic demonstrated that administration of a single dose (20 μg/g body weight) caused marked increases in serum PTH, but did not normalize serum calcium, whereas longer‐term administration of this dose induced normocalcemia in ADH1 mice.^(^ ^24^ ^)^

NPSP795 also increased plasma phosphate concentrations in WT and *Casr* ^*+/Nuf*^ mice, and such effects have previously been observed following administration of the NPS 2143 calcilytic compound to *Nuf* mice and an ADH2 mouse model, which harbors a gain‐of‐function Gα_11_ mutation, Ile62Val.^(^ ^26^, ^35^ ^)^ The cause of the increase in phosphate is unclear, as calcilytic treatment would be expected to lower plasma phosphate by inducing PTH‐mediated renal phosphate excretion.^(^ ^8^ ^)^ In keeping with this, ADH1 mice harboring a CaSR mutation, Cys129Ser, showed a decrease in serum phosphate concentrations following treatment with the JTT‐305/MK‐5442 calcilytic compound.^(^ ^24^ ^)^ The hyperphosphatemia observed in the current study may potentially have arisen because of dehydration and decreased renal function caused by the acute rise in plasma calcium following NPSP795 treatment, which may activate the kidney CaSR, thereby leading to polyuria.^(^ ^36^ ^)^ In support of this, WT and *Nuf* mice had elevations of plasma urea, which accompanied the rise in plasma calcium following NPSP795 treatment (Fig. 3). Moreover, the physiological stress associated with drug administration and blood sampling may have reduced water intake in the mice, thus exacerbating the dehydration and consequent increase in plasma urea. However, the increase in plasma urea concentrations appeared to be transient and had normalized in WT and *Casr* ^*+/Nuf*^ mice by 6 hours postdose (Fig. 3).

A limitation of this study is that despite *Casr* ^*Nuf/Nuf*^ mice being viable,^(^ ^28^ ^)^ fewer homozygotes were born than expected. Thus, the range of NPSP795 doses and study time points, which could be evaluated in *Casr* ^*Nuf/Nuf*^ mice, were limited. However, the reduced numbers of *Casr* ^*Nuf/Nuf*^ mice did not affect the main objective of this study, which was to evaluate NPSP795 in *Casr* ^*+/Nuf*^ mice, as this mouse genotype is a model for patients with ADH1 harboring germline heterozygous gain‐of‐function CaSR mutations. Furthermore, our study only evaluated the effect of NPSP795 on the in vitro and in vivo consequences of a single gain‐of‐function CaSR mutation. However, it is likely that this calcilytic will be of benefit for a range of ADH1‐causing CaSR mutations. Consistent with this, NPSP795 has been previously shown to improve the gain‐of‐function caused by mutations located in the extracellular (Glu228Ala, Glu228Lys, and Gln245Arg) and transmembrane (Ala840Val) domains of the CaSR.^(^ ^23^ ^)^

In conclusion, single‐dose administration of NPSP795 has been shown to cause dose‐dependent increases in PTH and to ameliorate the hypocalcemia in an ADH1 mouse model. Thus, the NPSP795 calcilytic represents a potential targeted therapy for ADH1. Longer‐term dosing studies are required to investigate whether NPSP795 can rectify the hypocalcemia caused by ADH1.

## Disclosures

FMH and RVT have received grant funding from NPS/Shire Pharmaceuticals and GlaxoSmithKline for studies involving the use of calcilytic compounds. RVT has received grants from Novartis Pharma AG and the Marshall Smith Syndrome Foundation for unrelated studies.

## Author Contributions

**Fadil Hannan:** Conceptualization; formal analysis; methodology; project administration; writing‐original draft; writing‐review and editing. **Caroline Gorvin:** Formal analysis; investigation; methodology; writing‐original draft; writing‐review and editing. **Valerie Babinsky:** Methodology; writing‐review and editing. **Mie Olesen:** Formal analysis; writing‐review and editing. **Michelle Stewart:** Investigation; project administration; writing‐review and editing. **Sara Wells:** Conceptualization; project administration; writing‐review and editing. **Roger Cox:** Conceptualization; methodology; writing‐review and editing. **Ed Nemeth:** Conceptualization; methodology; writing‐review and editing. **Rajesh Thakker:** Conceptualization; funding acquisition; supervision; writing‐original draft; writing‐review and editing.

### Peer Review

The peer review history for this article is available at https://publons.com/publon/10.1002/jbm4.10402.

## Data Availability

All data will be made available in a public repository such as Figshare after publication of the article.
